# Professor Bang’s Views

**Published:** 1897-04

**Authors:** 


### PROFESSOR BANG'S VIEWS.

So much has been said on the public platform, so much has been published in the daily press relative to the conclusions reached upon bovine tuberculosis by Professor Bang in Denmark, that we deem it wise and just to all concerned to publish in this number a carefully prepared abstract by one who has for years studied the work and investigations of Professor Bang, and who has been in correspondence with him, and is therefore well fitted to quote his views accurately and thus enable our readers to better answer many of those who are quoting Professor Bang in such a way as to unfairly and improperly influence the minds of many who are seeking light on this subject through the veterinarian.

